# THE EFFECT OF COMMUNITY HEALTHCHOICES ON PARTICIPANT SATISFACTION WITH HOME AND COMMUNITY-BASED SERVICES

**DOI:** 10.1093/geroni/igac059.565

**Published:** 2022-12-20

**Authors:** Todd Bear

**Affiliations:** University of Pittsburgh, Pittsburgh, Pennsylvania, United States

## Abstract

A stratified random sample of participants was interviewed in each region of Pennsylvania during each phase of the implementation using the Consumer Assessment of Health Providers Survey – Home and Community Based Services (CAHPS-HCBS) version. In addition, comparison groups were interviewed from the third implementation region. These data were combined with surveys conducted by the three Community HealthChoices managed care organizations (MCOs). Data were weighted to create geographically representative estimates of participant experience across a range of composite measures. We found that participant ratings of their personal care attendants declined on three out of four measures, however measures of medical and non-medical transportation improved as a result of the implementation of Community HealthChoices. There were notable differences between people of different racial and ethnic groups, with non-Hispanic whites consistently reporting lower levels of satisfaction with person care.

